# Concordance between TIRADS and Cytology in Thyroid Nodule

**DOI:** 10.22038/ijorl.2022.61654.3119

**Published:** 2022-11

**Authors:** Amin Abolhasani Foroughi, Maral Mokhtari, Emad Heidari, Masoume Nazeri, Hemmat Rastgouyan, Amirhossein Babaei

**Affiliations:** 1 *Medical Imaging Research Center, Department of Radiology, Shiraz University of Medical Science, Shiraz, Iran.*; 2 *Pathology department, Shiraz University of Medical Science, Shiraz, Iran.*; 3 *Clinical Neurology Research Center,* *Shiraz University of Medical Science Shiraz, Iran.*; 4 *Otolaryngology Research Center, Department of Otolaryngology, Shiraz University of Medical Sciences, Shiraz, Iran.*; 5 *Student Research Committee, Shiraz University of Medical Sciences, Shiraz, Iran.*

**Keywords:** Fine-Needle aspiration, Thyroid nodule, Ultrasonography, TI-RADS, Biopsy.

## Abstract

**Introduction:**

Palpable thyroid nodules are stated in 4 to 7% of individuals. This study was designed to evaluate the relation of Thyroid Imaging Reporting and Data System (TIRADS) and fine-needle aspiration (FNA) based cytology reports in patients with thyroid nodules.

**Materials and Methods:**

In this retrospective cross-sectional study, individuals with thyroid nodules who were selected for ultrasonographic-guided FNA enrolled in this study. Demographic data, radiologic assessment, and cytology report were gathered based on hospital medical records. TIRADS grading of the nodules was assessed for each nodule. Cytology was performed on all samples. Sensitivity and specificity were calculated by comparing cytology with ACR-TIRADS and also cytology with TIRADS 4-5 cut-off point as a radiologic malignant lesion.

**Results:**

172 patients were studied, 151 of whom were female and 21 were male. The mean age of the patients was 49.46 years. Most of the patients had TIRADS 4 (53.5%) followed by 3 (31.4%), and 5 (11.6%). 151 patients (87.8%) had a benign lesion in cytology. Of them, 118 had colloid nodules. There was a statistically significant relation between TIRADS and cytology (p-value<0.001). Sensitivity, specificity, AUC, and positive and negative predictive value for ACR-TIRADS classification were 76.19%, 47.54%, 0.619, 20.00%, and 92.06%, respectively. These values for cut-off “4-5” classification was 86.36%, 38.00%, 0.622, 16.96%, and 95.00%.

**Conclusions:**

According to the significant concordance between TIRADS and cytology, as shown in the results of our study, it seems that TIRADS could be used to decrease the amount of unnecessary FNA in individuals with thyroid nodules.

## Introduction

Palpable thyroid nodules are stated in 4% to 7% of individuals (1). About 5% of palpable nodules are malignant (2). Numerous guidelines have been released to ameliorate the diagnosis of thyroid nodules and their treatment, as well as improve the prognosis of the patients (3). Accurate prediction of malignant and non-malignant thyroid nodules is a challenging issue. Pathologic findings are currently the gold standard for the differentiation of non-malignant from malignant lesions. As thyroid fine-needle aspiration (FNA) is an invasive procedure, the determination of prognosis based on radiologic findings is currently taken into consideration (4). Horvath et al. have described Thyroid Imaging Reporting and Data System (TIRADS) classification based on an ultrasound study. They predicted the rate of malignancy in each group as follows: 0% for TIRADS 1 and 2, less than 5% for TIRADS 3, 5%-10% for TIRADS 4A, 11%-65% for TIRADS 4B, 66%-95% for TIRADS 4C, more than 95% for TIRADS 5, and proved malignancy in TIRADS 6 (5, 6). In a retrospective study, Singaporewalla et al. compared the results of TIRADS and FNA cytology described by the Bethesda System in 100 cases. They reported a concordance rate of 83%. Sensitivity, specificity, and negative predictive values were 70.6%, 90.4%, and 93.8% (7). Another study by Vargas-Uricoechea et al. determined the association between TIRADS and cytopathology in 180 patients. They found that TIRADS had an acceptable concordance with the Bethesda system (8). Other studies confirmed these findings (9, 10). As a result of the efficacy of the “American College of Radiology’s breast imaging reporting and data system” (BIRADS) classification for breast lesions (11), Horvath et al. developed a new TIRADS for thyroid nodules (6).

In BIRADS classification, a puncture will be done for nodules with BIRADS 4 and 5, and the lesions in the rest of the categories will follow up. Horvath et al. compared TIRADS with the pathology reports in 210 patients who had 502 surgically removed nodules. The researchers found the risk of malignancy among TIRADS 2, 3, 4, and 5 at 0%, 1.79%, 76.13%, and 98.85%, respectively. Using TIRADS 4-5 as a cut-off point to perform FNA, they stated a 99.6% sensitivity and 74.35% specificity (6). The purpose of the current investigation was to compare the results of TIRADS and cytology reports in patients with thyroid nodules.

## Materials and Methods

This retrospective cross-sectional investigation was conducted on patients with thyroid nodules who were referred to Faghihi Hospital affiliated to Shiraz University of Medical Sciences, Shiraz, Iran, for FNA under the guide of sonography. The inclusion criteria were patients who had thyroid nodules either detected by palpation and referred by a clinician or detected in ultrasonography. On the other hand, patients with previously diagnosed thyroid cancer were excluded from the study.

Before conducting the study, the protocol was assessed and approved by the Ethics Committee of Shiraz University of Medical Sciences, Shiraz, Iran (ethic code: IR.sums.med.rec.1398.86). The study was performed according to the principles of the Declaration of Helsinki. Each participant signed a printed informed consent form on admission to permit using the information of their medical records with consideration of their privacy.

Demographic characteristics (age and gender), ultrasonographic assessment (echogenicity, tumor border, tumor size, composition, orientation, and calcification), and cytology report were gathered from documented hospital medical records.

Lesions were categorized according to TIRADS which were described previously (5). Ultrasound scanner WS80A (Samsung Medison Ltd, Korea) with 5-12 linear transducers was used. TIRADS grading, echogenicity, margin, shape, tumor size, composition, and calcification of the nodules were recorded for each nodule by a skillful radiologist with more than 10 years of practice in doing ultrasonography by himself. 

After the confirmation of thyroid nodule by ultrasound, FNA was performed under the guidance of ultrasound with an 18-gauge needle attached to a 20-ml disposable plastic syringe. Ultrasound-guided FNA was done in either the thyroid nodule with doubtful ultrasound characteristics or the greatest thyroid nodule with the same TIRADS feature among other nodules. Materials collected from FNA were thrown out on 6 glass slides and smeared. Three air-dried slide smears and three Pathofix sprays (Padtan Teb Company, Tehran, Iran) fixed slides were evaluated at the pathology laboratory. Cytology was performed on all samples. The FNA reports were categorized according to the Bethesda system 2017 (12) and recorded for each nodule separately.

To perform statistical analysis, the lesions were classified as benign and non-benign. Non-benign lesions included AFLUS (atypical follicular lesion of undetermined significance), follicular neoplasm or suspicious for a follicular neoplasm, suspicious for malignancy, and malignant. Sensitivity, specificity, area under the curve (AUC), negative and positive likelihood ratio, positive and negative predictive value, and accuracy were measured by comparing cytopathologic findings with the American College of Radiology Thyroid Imaging Reporting and Data System (ACR-TIRADS) guideline (13) or consideration of TIRADS 4-5 cut-off point as a radiologic malignant lesion (6). The data were analyzed by SPSS software (version 25, SPSS Inc., Chicago, IL, USA). Chi-square tests were also applied to determine the significance of the relationship between the TIRADS score and cytology reports. A P-value of ≤0.05 was considered statistically significant.

## Results

In total, 172 patients were investigated in this study, and the majority of the cases (n=151; 87.8%) were female. The mean±SD age of the patients was obtained at 49.46±13.86 years. Most of the patients had TIRADS 4 (53.5%), followed by 3 (31.4%) and 5 (11.6%). Radiologic findings of thyroid nodules are shown in Table 1. 

**Table 1:** Radiologic findings of the patients with thyroid nodules

**Variable**	**Category**	**Number (%)**
TIRADS grading	2	6 (3.5%)
	3	54 (31.4%)
	4	92 (53.5%)
	5	20 (11.6%)
Echogenicity	Hyperechoic	11 (6.4%)
	Hypoechoic	108 (62.8%)
	Isoechoic	43 (25%)
	Very hypoechoic	10 (5.8%)
Margin	Ill-defined	6 (3.5%)
	Irregular	4 (2.3%)
	Lobulated	6 (3.5%)
	Smooth	156 (90.7%)
Shape	Wider than tall	172 (100%)
	Taller than wide	0 (0%)
Tumor size	Less than 1 cm	27 (15.7%)
	1 to 1.5 cm	30 (17.4%)
	1.5 to 2.5 cm	35 (20.3%)
	Equal or more than 2.5 cm	51 (39.7%)
	Missing	29 (16.9%)
Composition	Solid	117 (68.0%)
	Solid cystic	54 (31.4%)
	Spongiform	1 (0.6%)
Echogenic foci	Macrocalcification	27 (15.7%)
	None	97 (56.4%)
	Peripheral calcification	11 (6.4%)
	Punctate calcification	37 (21.5%)

A total of 151 (87.8%) patients had a benign lesion in the cytology. Of these, 118 cases had colloid nodules. Table 2 tabulates the detailed cytology report of the patients with thyroid nodules. According to Table 3, there is a statistically significant relationship between TIRADS and cytology (P<0.001).

**Table 2:** Cytology of the patients with thyroid nodules

**Category**	**Number**	**Percent**
Nondiagnostic or unsatisfactory	8	4.7%
Benign	142	82.6%
Atypia of undetermined significance or follicular lesion of undetermined significance	12	7.0%
Follicular neoplasm or suspicious for a follicular neoplasm	1	0.6%
Suspicious for malignancy	6	3.5%
Malignant	3	1.7%

**Table 3:** Concordance between TIRADS and cytology

**TIRADS**	**Cytology**			**p-value**
	**Benign**	AFLUS*	**Malignant**	
2	5 (3.3%)	1 (8.3%)	0 (0.0%)	<0.001 ^
3	52 (34.7%)	2 (16.7%)	0 (0.0%)	
4	82 (54.7%)	6 (50.0%)	4 (40.0%)	
5	11 (7.3%)	3 (25.0%)	6 (60.0%)	

* Atypical follicular lesion of undetermined significance ^ Fisher's exact test

**Fig 1:**
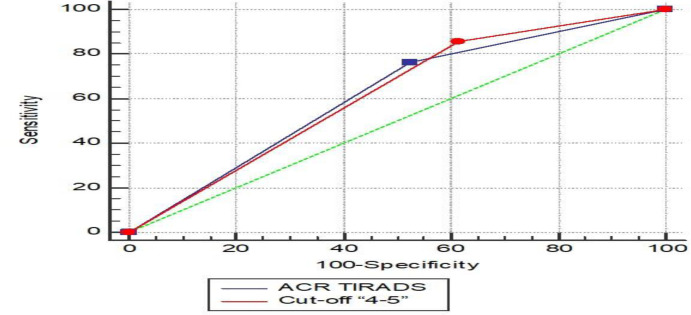
Comparison of AUC in ROC curve in ACR-TIRADS and Cut-off “4-5”

Sensitivity, specificity, AUC, positive predictive value, and negative predictive value for ACR-TIRADS classification were estimated at 76.19%, 47.54%, 0.619, 20.00%, and 92.06%, respectively. These values for cut-off “4-5” classification were determined at 86.36%, 38.00%, 0.622, 16.96%, and 95.00% (Figure 1 and Table 4).

**Table 4:** Comparison of ACR-TIRADS and Cut-off “4-5”

**Parameter**	ACR-TIRADS*	**Cut-off “4-5”**
Sensitivity	76.19%	86.36%
Specificity	47.54%	38.00%
The area under the curve (AUC)	0.619	0.622
Positive likelihood ratio	1.452	1.393
Negative likelihood ratio	0.501	0.359
Positive predictive value (PPV)	20.00%	16.96%
Negative predictive value (NPV)	92.06%	95.00%
Accuracy	51.75%	44.19%

* American College of Radiology Thyroid Imaging Reporting and Data System

## Discussion

This study investigated the concordance between ACR-TIRADS and cytology reports in patients with a thyroid nodule. The incidence of thyroid nodules is still high and has been reported from 50% to 60% in healthy subjects (14). About 90% of thyroid nodules are benign and 95% are without any symptoms (15). FNA is the gold standard technique for differentiating benign from malignant nodules (3). Approximately, 8%-20% of FNAs are insufficient for diagnosis, and repetition is needed (16). Extensive use of imaging techniques, especially ultrasound, has increased the detection rate of thyroid nodules. The main purpose of thyroid ultrasound is to differentiate benign from suspected malignant nodules. Some studies showed that the size of the nodule is an important factor in the determination of the risk of malignancy (17). On the other hand, the location of the nodule was an independent risk factor for malignancy (18). The main disadvantage of ultrasound is its relatively low specificity and dependence on the experience of the operator (19). To minimize these limitations, some efforts have been made to develop and modify a standard system for the classification of thyroid nodules. Three of these guidelines are made by surgeons, endocrinologists, and radiologists, respectively, published by the American Thyroid Association (ATA) in 2015 (20), the American Association of Clinical Endocrinologists, the American College of Endocrinology, the Association Medici Endocrinology (AACE/ACE/AME) Guidelines in 2016 (14), and ACR in 2017 (13). The ultrasound characteristics and nodule size cut-off point for FNA differ among these guidelines. This has led to differences in the amount of performed FNA and the rate of malignancy detection (21).

According to ATA, FNA is essential for a solid hypoechoic nodule or solid hypoechoic component of a partially cystic nodule ≥1 cm with irregular margins, microcalcifications, taller than wide shape, rim calcifications with small extrusive soft tissue component, evidence of extrathyroidal extension. Furthermore, FNA is suggested for isoechoic or hyperechoic solid nodule, or partially cystic nodule size more than 1.5 cm with eccentric solid areas, without microcalcification, irregular margin, or extrathyroidal extension, or taller than wide shape (20). According to AACE/ACE/AME Guideline, FNA is indicated for ultrasonic high-risk thyroid lesions with a size of more than 10 mm, intermediate-risk lesions >20 mm, low-risk lesions >20 mm and increasing in size or accompanying with high-risk history, prior to thyroid operation and minimally invasive ablation therapy (14).

ACR-TIRADS classification was first introduced by Horvath et al. (5). Since its introduction, numerous types of TIRADS have been proposed (22), such as British Thyroid Association (BTA) (23), Kwak- TI-RADS (24), and the European Thyroid Association (EU-TIRADS) (25). The variety of sonographic characteristics that are highly prognostic for malignancy in different guidelines increases the need for the experience of physicians (26). In the present study, about 88% of the study population was female, which is consistent with other studies that report a higher occurrence of thyroid nodules in females. In addition, the mean age of the patients was 49.46 years, which is in line with a higher incidence of thyroid nodules in adults (27). In our study, most patients were in grades 3 and 4A of TIRADS and had benign thyroid nodules. In a study, Yoon et al. investigated the risk of malignancy of thyroid nodules. Of 1293 thyroid nodules, 1059 (81.9%) were benign and 234 (18.1%) were malignant. Malignancy rates of TIRADS 3, 4A, 4B, 4C and 5 were 1.9%, 4.2%, 12.9%, 49.8%, and 92.3%, respectively (28). Furthermore, a study by Horvath et al. evaluated and validated the classification of TIRADS for resected thyroid nodules. They showed that ultrasound-based TIRADS can differentiate malignant from benign nodules (6). The result of this study is in line with our findings.

Wildman-Tobriner et al. used artificial intelligence to improve ACR-TIRADS. In this study, ACR-TIRADS with a single expert reader showed sensitivity and specificity of 93.3% and 47.1%. The AUC for ACR-TIRADS was 0.91 (29). In another study, sensitivity (94.0%), specificity (28.2%), positive predictive value (37.3%), and negative predictive value (91.2%) were calculated by considering TIRADS 4-5 as malignant and TIRADS 1-3 as benign (30). Fernández et al. also used cut-off TIRADS 4-5 as malignant. The sensitivity in their study was 65% (31). In another study by Clark et al., the sensitivity and specificity of ACR-TIRADS were 85% and 38.6%, respectively (32). In another study published by Sahli et al., the concordance of TIRADS and pathological results were examined. The results showed that the sensitivity, specificity, positive predictive value, negative predictive value, and accuracy of ACR-TIRADS were 71.4%, 38.1%, 40.2%, 69.6%, and 50.4%, respectively (33).

Regarding the limitations of our study, one can refer to the retrospective method as the most important problem. Another issue was related to the histopathological assessment since it was not possible to do it for patients with cytologic confirmed benign nodules. On the other hand, the advantage of our study is the calculation and comparison of sensitivity, specificity, and other variables based on both ACR-TIRADS and TIRADS 4-5 cut-off points. It is suggested that the researchers perform further studies with a prospective design and larger sample sizes.

## Conclusion

This study established the value of the concordance between the ACR-TIRADS and cytology. According to reported sensitivity, specificity, and other parameters, there is a significant concordance between ACR-TIRADS and cytology. It seems that TIRADS could be used to decrease unnecessary FNA for possible benign-looking nodules.
